# Detection of S^2^^−^ in Water by a Glucose Enhanced Water-Soluble Fluorescent Bioprobe

**DOI:** 10.3390/bios12080600

**Published:** 2022-08-04

**Authors:** Xingwang An, Yi Wang, Jiahui Li, Zhichao Pei, Yuxin Pei

**Affiliations:** Shaanxi Key Laboratory of Natural Products and Chemical Biology, College of Chemistry & Pharmacy, Northwest A&F University, Yangling, Shaanxi 712100, China

**Keywords:** bioprobe, fluorescent test strips, sulfide anion, water solubility, carbohydrate

## Abstract

That sulfide anions (S^2−^) in aquatic environments are produced by microorganisms through degrading sulfur-containing proteins and other organics are harmful to human health. Thus, it is of significance to develop a convenient method for the detection of S^2−^ in water. Small molecular fluorescent probes are very popular for their advantages of visualization, real-time, high sensitivity, and convenience. However, low solubility in water limits the application of existing S^2−^ probes. In this work, we found that our previously developed water-soluble glycosylated fluorescent bioprobe Cu[GluC] can achieve detection of S^2−^ in water. Cu[GluC] can restore fluorescence within 20 s when it encounters S^2−^ and shows good sensitivity towards S^2−^ with a detection limit of 49.6 nM. Besides, Cu[GluC] derived fluorescent test strips were obtained by immersion and realized conveniently visual S^2−^ detection in water by coupling with a UV lamp and a smartphone app. This work provides a fluorescent bioprobe with good water solubility as well as its derived fluorescent test strip for sensitive and simple detection of S^2−^ in water, which shows good prospects in on-site water quality monitoring.

## 1. Introduction

S^2−^ is a common environmental pollutant, which comes from the metabolites of microbial decomposition of organic matter [1,2]. S^2−^ in environmental pollution can pose a threat to the health of animals, plants and even humans [3]. It is of significance to develop a convenient method for the detection of S^2−^ in water.

Among different kinds of detection methods, small molecular fluorescent probes are very popular for their advantages of visualization, real-time, high sensitivity, and convenience [4,5]. At present, there are three main strategies to construct fluorescent probes for detecting S^2−^: the metal displacement strategy, azide reduction strategy, and nucleophilic reaction strategy [6,7,8]. Among them, fluorescent probes for the detection of S^2−^ constructed by the metal ion displacement strategy have attracted much attention due to their high selectivity and short response time. Various fluorescent probes based on the Cu^2+^ displacement strategy have been reported successively. They are mostly derivatives of common fluorophores, such as coumarin, fluorescein, phthalazine-imidazole and BODIPY [6,9,10,11]. However, there is a problem that most fluorescent probes lack water solubility [12,13,14,15], which limits their application in S^2−^ detection in water. A few water-soluble fluorescent probes have been reported by introducing hydrophilic groups, such as benzyl carbazate, phenolic, and trihydroxyl groups [6,11,16]. But they still have disadvantages of a narrow pH range, or complicated synthetic routes. Therefore, it is necessary to develop a water-soluble bioprobe for sensitive and rapid S^2−^detection in water.

**Scheme 1:**
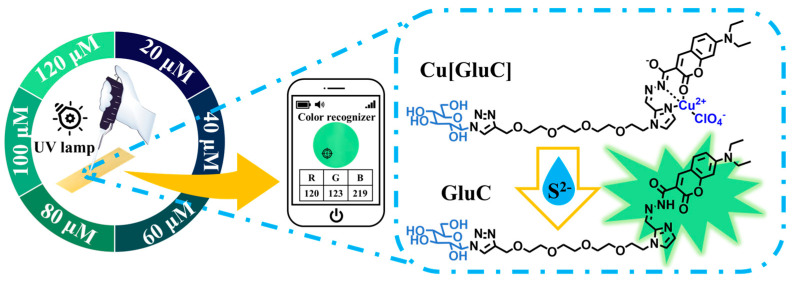
Detection of S^2−^ in water by a glucose enhanced water-soluble fluorescent bioprobe.

Carbohydrates are abundant biological molecules in nature with good water solubility and biocompatibility. They are widely used in the modification of drugs, probes and biomaterials to enhance their water solubility [17,18,19,20,21]. Introducing carbohydrates may be a good choice for improving the water solubility of S^2−^ fluorescent probes. Here, we found that our previously developed glycosylated fluorescent bioprobe Cu[GluC] can achieve S^2−^ detection in water with a low detection limit. The fluorescence of Cu[GluC] can recover quickly within a few seconds when encountering S^2−^ in water based on the metal ion displacement strategy. Fluorescent test strips produced by Cu[GluC] displayed obvious color changes under 365 nm UV light after dropping S^2−^ aqueous solution with different concentrations, which can achieve simply S^2−^ detection by coupling with a UV lamp and a color recognizer app in a smartphone (Scheme 1). It is important to emphasize that this bioprobe Cu[GluC] and its derived fluorescent test strips realize convenient and sensitive S^2−^ detection in water, which shows application prospects for water quality monitoring. To the best of our knowledge, enhancing water solubility of the fluorescent probe by introducing a glucose unit for the detection of S^2−^ has not been reported yet.

## 2. Materials and Methods

### 2.1. Reagents and Instruments

Reagents related to the synthesis of Cu[GluC] were commercial reagents without further purification and were provided in the Supplementary Material. The water used in this work was ultrapure water. Sodium, potassium, and copper salts were purchased from Adamas Chemical Reagent Co. Stock solutions of these salts were prepared with ultrapure water, which was also used throughout the study. The synthetic compounds were characterized with a nuclear magnetic resonance (NMR) spectrometer (Bruker, Karlsruhe, Germany) where the residual signals from DMSO-*d*_6_ (^1^H: *δ* 2.50 ppm) or Chloroform-*d* (^1^H: *δ* 7.26 ppm) were used as internal standards and High-resolution ion mobility liquid chromatography–mass spectrometry (HRLCMS) (LC-30A + TripleTOF5600+, AB SCIEX, USA). The UV-vis spectra were measured with a 1750 UV-visible spectrophotometer (Shimadzu, Japan), and the fluorescence spectra were measured with an RF-6000 fluorescence spectrophotometer (Shimadzu, Kyoto, Japan).

### 2.2. Synthesis of Cu[GluC]

**Scheme 2:**
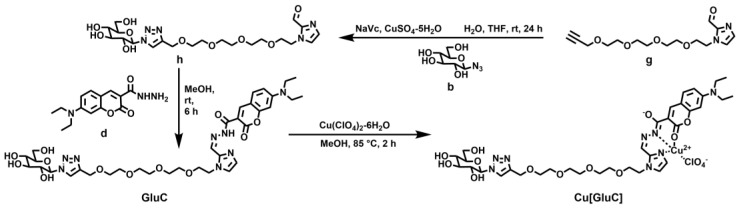
Synthesis of GluC and Cu[GluC].

As shown in Scheme 2, compound **g** reacted with compound **b** to obtain compound **h** through a ‘click’ reaction and compound **h** reacted with compound **d** to obtain GluC through a Schiff base reaction. Finally, GluC reacted with Cu(ClO_4_)_2_·6H_2_O to obtain Cu[GluC]. The detailed synthesis route was provided in Supplementary Material according to our previous study [22] with some changes. ^1^H NMR and HRMS of the synthesized compounds were provided in Figures S1–S10.

### 2.3. Procedures of the Ion Sensing

The stock solution of GluC, Cu[GluC] and related substrates were prepared in ultrapure water. The samples were added in a 1 cm quartz cuvette and then measured with a UV-visible spectrophotometer and a fluorescence spectrophotometer. The excitation is carried out at 450 nm, and the slit width of emission and excitation is 5 nm. Besides, the calculation of quantum yield (Φ), binding constant (*K_α_*), dissociation constant (*K_d_*) in the experiment refers to the methods provided by our previous study [22]. Limit of detection (LOD) and limit of quantification (LOQ) are calculated as follows: “LOD = 3 σ/k”, “LOQ = 10 σ/k”, where σ is the standard deviation of blank and k is the linear correlation slope of fluorescence intensity at 494 nm relative to the concentrations of S^2−^.

### 2.4. Preparation of Test Strips

Filter papers were dipped in Cu[GluC] aqueous solution (100 μM) for 30 min and dried naturally to make fluorescent test strips. A procedure for fluorescent test strip analysis is presented. First, 10 μL S^2−^ aqueous solution with different concentrations (20–120 μM) were dropped onto the fluorescent test strips that were prepared. After two minutes, photos of test strips were taken by a smartphone (PBBM30, OPPO, Dongguan, China) under a UV light of 365 nm. Then, RGB (red, green and blue) values of fluorescent test strips were obtained via a color recognizer app (V8.100, Xiyi Technology, Xiamen, China).

## 3. Results and Discussion

### 3.1. The Coordination Mode between GluC and Cu^2+^

**Figure 1:**
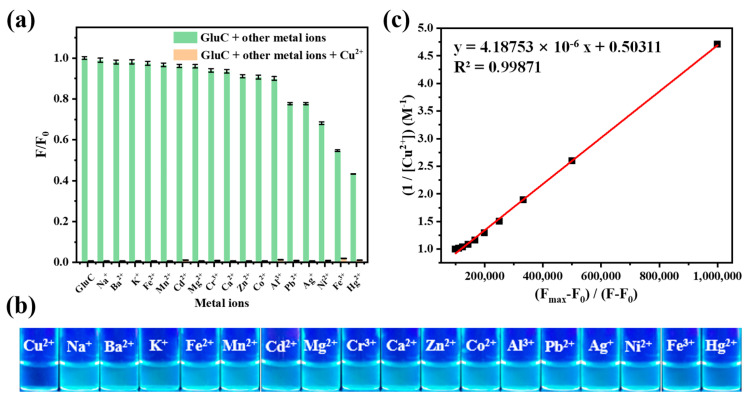
(**a**) The influence of different metal ions (20.0 μM) to fluorescence intensity of GluC (10.0 μM) at 494 nm followed by adding Cu^2+^ (20.0 μM) in water (*n* = 3, “*n*” means number of experiment repeats); (**b**) Visible color changes of GluC (10.0 μM) under UV light (365 nm) when adding various metal ions (20.0 μM); (**c**) The Benesi-Hilderbrand plots of GluC with Cu^2+^.

The binding specificity of GluC to Cu^2+^ under the influence of different metal ions was first investigated. In comparison to other metal ions, only Cu^2+^ caused the maximum fluorescence quenching of GluC (Figure 1a,b). In addition, the complexation of GluC with Cu^2+^ was not affected by other metal ions. The *K_α_* of GluC-Cu^2+^ (Cu[GluC]) was calculated to be 2.388 × 10^5^ M^−1^ with a good linear relationship (R^2^ = 0.9989) according to Benesi–Hilderbrand equation (Figure 1c). The fluorescence quantum yield of Cu[GluC] before and after responding to S^2−^ (2 equiv.) in an aqueous solution was 0.018 and 0.104. What is more, according to our previous study [22] and a Job’s plot (Figure S12), GluC and Cu^2+^ have formed the 1:1 complex. Besides, a peak of Cu[GluC] was found at 834.2602 in the mass spectrum (Figure S10), which is corresponding to [GluC + Cu^2+^]. Thus, all the above results suggest that the binding ratio of GluC and Cu^2+^ is 1:1. The *K_d_* for Cu[GluC] was calculated to be 2.033 μM in our previous study [22], which indicates the good stability of Cu[GluC].

### 3.2. S^2−^ Responsiveness of Cu[GluC]

**Figure 2:**
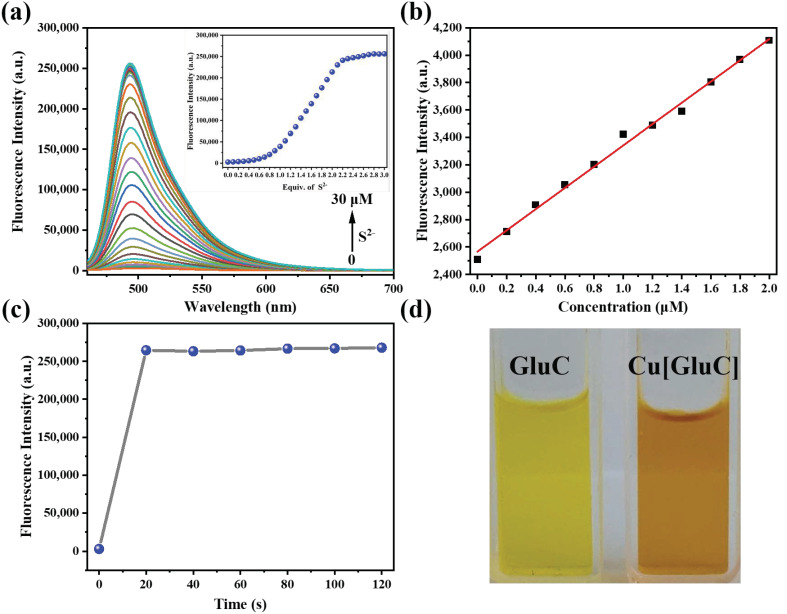
(**a**) The fluorescence spectra titration of Cu[GluC] (10.0 μM) with increasing concentrations of S^2−^ in aqueous solution. Inset: Fluorescence intensity changes of Cu[GluC] at 494 nm with the increasing of S^2−^concentrations^−^; (**b**) The linear relationship of the fluorescent intensity (494 nm) of Cu[GluC] (10 μM) relative to the concentrations of S^2−^ in aqueous solution; (**c**) The fluorescence variation process (494 nm) of Cu[GluC] after adding S^2−^ in aqueous solution within 120 s; (**d**) The aqueous solution of Cu[GluC] and GluC under visible light.

The spectroscopic responses of Cu[GluC] towards S^2−^ were studied. As shown in Figure 2a, the fluorescence intensity of Cu[GluC] gradually increased with the addition of S^2−^ and reached the plateau after adding 2.0 equiv of S^2−^. Fluorescence intensity shows a good linear relationship (R^2^ = 0.9941) towards the concentration of S^2−^ ranging from 0–2 μM, according to which, the LOD and LOQ were calculated to be 49.6 nM and 165.3 nM, respectively (Figure 2b). Compared with other fluorescent probes [23,24,25,26], Cu[GluC] has a lower detection limit, indicating that Cu[GluC] has a sensitive detection ability toward S^2−^ in water. The fluorescence responsive speed of Cu[GluC] (10.0 μM) towards S^2−^ (30.0 μM) in water was further tested. As shown in Figure 2c, after adding S^2−^ into aqueous solutions of Cu[GluC], the fluorescent intensity of Cu[GluC] increased quickly and reached the plateau within 20 s, which is faster than other S^2−^ fluorescent probes [23,27,28,29], suggesting the quick S^2−^ detection ability of Cu[GluC]. 

### 3.3. pH Stability and Water Solubility of Cu[GluC] and GluC

The stability of a fluorescent probe in working conditions with a wide range of pH is vital. Therefore, the fluorescence stability of Cu[GluC] and GluC were studied in water with different pHs. According to our previous study [22], both Cu[GluC] and GluC displayed excellent stability in the pH range of 6–11, which is better than other S^2−^ fluorescent probes [23,28,30,31]. This result indicates that Cu[GluC] is suitable for the S^2−^detection in water with a wide pH range from 6 to 11. From the curve about pH influence on the fluorescence intensity of Cu[GluC], the pK_a_ is calculated to be 4.18. In addition, the aqueous solution of GluC and Cu[GluC] was clear and transparent even if the concentrations were 1 mM (Figure 2d), which suggests that they have excellent water solubility. What is more, all the fluorescence intensity and UV absorbance of the Cu[GluC] aqueous solution before and after adding S^2−^ were unchanged within 30 min (Figure S11), which suggests that Cu[GluC] has excellent solution stability in the presence/absence of the analyte.

### 3.4. Selectivity and Interference

**Figure 3:**
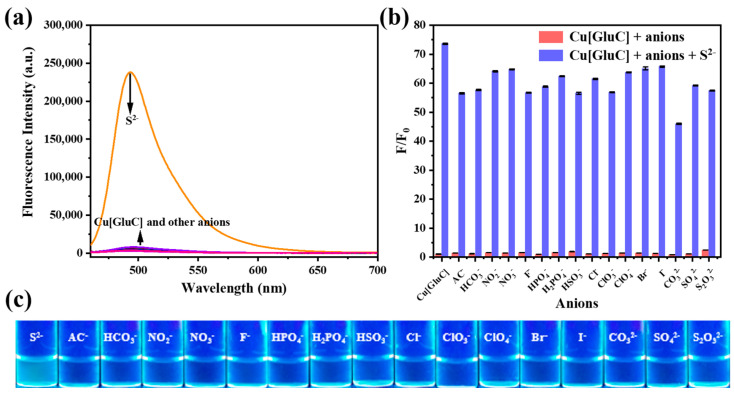
(**a**) Fluorescence emission spectra of Cu[GluC] aqueous solution (10.0 μM) after adding different anions (30.0 μM); (**b**) Fluorescence intensity of Cu[GluC] aqueous solution (10.0 μM) at 494 nm in the presence of different anions (30.0 μM) and then adding S^2−^ (30.0 μM) in water (*n* = 3); (**c**) The fluorescence of Cu[GluC] aqueous solution (10 μM) under UV light (365 nm) after adding different anions (30.0 μM).

As we all know, selectivity is an important criterion to evaluate the performance of fluorescent probes. Thus, the specific responsiveness of Cu[GluC] to S^2−^ in water was studied via fluorescence and colorimetric method. As shown in Figure 3a,c, Cu[GluC] recovery its fluorescence only in the presence of S^2−^. Compared to adding other anions, Cu[GluC] aqueous solution (10 μM) shows light-blue under 365 nm UV lamp after adding S^2−^. What is more, other anions cannot show an effect on the fluorescence recovery of Cu[GluC] (Figure 3b). All these results demonstrate that Cu[GluC] possesses the ability of selective S^2−^ detection in water.

### 3.5. Visual S^2−^ Detection of by Fluorescent Test Strips

**Figure 4:**
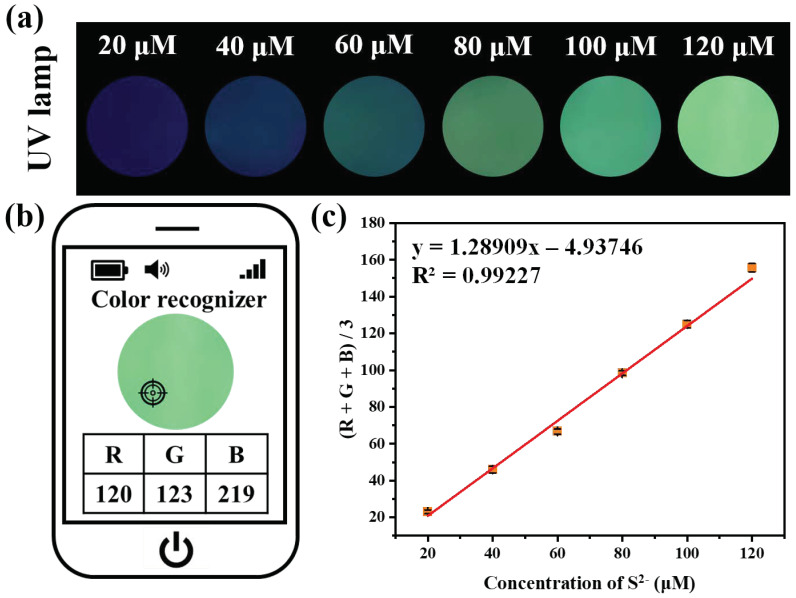
(**a**) Color changes of fluorescent test strips prepared by filter papers embedded with Cu[GluC] after treated with different concentrations of S^2−^ aqueous solution under UV lamp (365 nm) irradiation; (**b**) The RGB values of fluorescent test strips were obtained through a smartphone app; (**c**) The linear relationship of (R+G+B)/3 values of fluorescent test strips relative to the concentrations of S^2−^ (*n* = 3).

In order to make the S^2−^ detection in water more convenient, fluorescent test strips were simply fabricated by soaking filter paper into Cu[GluC] aqueous solution (100 μM) for 30 min and then air drying. Surprisingly, under 365 nm UV light, the color of fluorescent test strips changed significantly from blue-black to light green after treatment with different concentrations of S^2−^ (20–120 μM) (Figure 4a). Then, the RGB data of fluorescent test strips were acquired via a smartphone app (Figure 4b). As shown in Figure 4c, (R + G + B)/3 values had a good linear relationship with the concentrations of S^2−^ (R^2^ = 0.99227). All results demonstrate that the fluorescent test strips coupled with a portable UV lamp and a smartphone app can achieve quantitative and convenient on-site S^2−^ detection in water.

### 3.6. Comparison with Other Fluorescent Probes for S^2−^ Detection

A fluorescent probe for S^2−^ detection based on the Cu^2+^ replacement strategy has a broad application prospect at present. Because of the need for practical detection, improving water solubility is an important development direction for fluorescent probes for S^2−^ detection in water. The structures, properties, and highlights of some representative probes are listed in Table S1. Compared with other fluorescent probes for S^2−^ detection [16,23,32,33], Cu[GluC] not only achieves sensitive and selective S^2−^ detection in water with a wide pH range and short response time, but also its derived fluorescent test strips hold good potential in on-site water quality monitoring.

## 4. Conclusions

A small molecular fluorescent bioprobe Cu[GluC] with excellent water solubility endowed by a glucose unit was used for the detection of S^2−^ in water. Cu[GluC] can quickly and selectively respond to S^2−^ in water with a short response time, low detection limits and a wide pH range. Besides, fluorescent test strips can realize convenient on-site S^2−^ detection in water by coupling a UV lamp and a smartphone app. Thus, this work provides a fluorescent bioprobe with good water solubility and its derived fluorescent test strips for sensitive and convenient S^2−^ detection in water, which holds good potential in on-site water quality monitoring.

## Data Availability

The data presented in this study are available upon request from the corresponding author.

## Supplementary Materials

The following supporting information can be downloaded at: https://www.mdpi.com/article/10.3390/bios12080600/s1, Scheme S1: Synthesis of bioprobe Cu[GluC]; Figure S1: ^1^H NMR spectrum of compound a in Chloroform-*d*; Figure S2: ^1^H NMR spectrum of compound b in DMSO-*d*_6_; Figure S3: ^1^H NMR spectrum of compound c in Chloroform-*d*. Figure S4: ^1^H NMR spectrum of compound d in Chloroform-*d*; Figure S5: ^1^H NMR spectrum of compound e in Chloroform-*d*; Figure S6: ^1^H NMR spectrum of compound f in Chloroform-*d*; Figure S7: ^1^H NMR spectrum of compound g in Chloroform-*d*; Figure S8: ^1^H NMR spectrum of compound h in DMSO-*d*_6_; Figure S9: ^1^H NMR spectrum of GluC in Chloroform-*d*; Figure S10: HRMS spectrum of Cu[GluC]; Figure S11: The fluorescence intensity and absorbance variation process of Cu[GluC] and Cu[GluC] + S^2−^ in water within 30 min; Figure S12: Job’s plot for the stoichiometry of GluC and Cu^2+^ ions; Table S1: Some reported fluorescent probes for S^2−^ detection in water.
