# Supplementary material for: Detection of S2− in Water by a Glucose Enhanced Water-Soluble Fluorescent Bioprobe
Source: Biosensors (Basel). 2022 Aug 4;12(8):600. doi: 10.3390/bios12080600 (PMC9406183; doi:10.3390/bios12080600)
Supplement: Supplementary file 1 [file biosensors-12-00600-s001.zip › biosensors-1823666-supplementary.pdf]

biosensors

MDPI

Supporting Materials

Detection of S^{2-} in Water by A Glucose Enhanced Water-soluble Fluorescent Bioprobe

Xingwang An †, Yi Wang †, Jiahui Li, Zhichao Pei and Yuxin Pei *

Shaanxi Key Laboratory of Natural Products and Chemical Biology, College of Chemistry & Pharmacy, Northwest A&F University, Yangling 712100, China; sdlcsxaxw@nwafu.edu.cn (X.A.); wangyi060522@nwafu.edu.cn (Y.W.); ljh2021@nwafu.edu.cn (J.L.); peizc@nwafu.edu.cn (Z.P.)
* Correspondence: peiyx@nwafu.edu.cn
† These authors contributed equally to this work.

Citation: An, X.; Wang, Y.; Li, J.; Pei, Z.; Pei, Y. Detection of S^{2-} in Water by A Glucose Enhanced Water-Soluble Fluorescent Bioprobe. *Biosensors* **2022**, *12*, 600. https://doi.org/10.3390/bios12080600

Received: 3 July 2022
Accepted: 2 August 2022
Published: 4 August 2022

Publisher's Note: MDPI stays neutral with regard to jurisdictional claims in published maps and institutional affiliations.

Copyright: © 2022 by the authors. Submitted for possible open access publication under the terms and conditions of the Creative Commons Attribution (CC BY) license (https://creativecommons.org/licenses/by/4.0/).

1. Materials

All reagents were analytical grade unless otherwise noted and purchased from commercial suppliers and used without further purification. Water used in this work was ultrapure water. 2,3,4,6-tetra-O-acetyl-alpha-D-glucopyranosyl bromide, 4- (diethylamino)salicylaldehyde, diethyl malonate, sodium hydroxide, copper sulfate pentahydrate, copper(II) perchlorate hexahydrate and imidazole-2-carboxaldehyde were purchased from Adamas Chemical Reagent Co. Potassium carbonate, copper sulfate pentahydrate and acetic acid were purchased from Chron Chemical Reagent Co. Tosyl chloride, sodium methylate and sodium ascorbate were purchased from Aladdin Chemical Reagent Co. Sodium hydride and bis[2-(2-hydroxyethoxy)ethyl] ether were purchased from Xiya Chemical Reagent Co. Hydrazine monohydrate was purchased from J&K Chemical Reagent Co. 3-Bromopropyne was purchased from Jiuding Chemical Reagent Co.

2. Synthesis and characterization of Cu[GluC]

The detailed synthetic route and characterization about Cu[GluC] are described in Scheme S1 and Figure S1-S10.

Compound **a**: 2,3,4,6-tetra-O-acetyl-alpha-D-glucopyranosyl bromide (1.021 g, 2.5 mmol) and sodium azide (234 mg, 3.6 mmol) were added into dimethyl sulfoxide (10 mL). The reaction mixture was stirred for 0.5 hours at room temperature. Then the mixture was diluted with water (15 mL) and extracted with dichloromethane. The combined organic phase was washed with brine twice, dried over anhydrous Na_2SO_4 and evaporated to dryness to give colorless oil, yield 96%. ^1H NMR (400 MHz, Chloroform-*d*) δ 5.22 (t, J = 9.5 Hz, 1H), 5.11 (t, J = 9.7 Hz, 1H), 4.96 (t, J = 9.2 Hz, 1H), 4.65 (d, J = 8.9 Hz, 1H), 4.28 (dd, J = 12.5, 4.8 Hz, 1H), 4.21 – 4.12 (m, 1H), 3.80 (ddd, J = 10.0, 4.8, 2.3 Hz, 1H), 2.06 (dd, J = 28.1, 9.5 Hz, 12H) ppm.

Compound **b**: CH_3ONa (40 mg, 0.75 mmol) was added into the solution of **a** (201 mg, 0.5 mmol) in methanol (5 mL). The reaction mixture was stirred at room temperature for 2 hours and then neutralized by addition of ion-exchange resin (Amberlite IR 120 H^+) until pH = 7. The mixture was filtered and the solvent was evaporated to dryness to give colorless solid, yield 84%. ^1H NMR (400 MHz, DMSO-d_6) δ 5.66 – 5.25 (m, 2H), 4.74 (s, 1H), 4.45 (d, J = 8.6 Hz, 1H), 3.66 (dd, J = 11.9, 2.1 Hz, 1H), 3.44 (dd, J = 11.9, 5.7 Hz, 1H), 3.26 – 3.13 (m, 3H), 3.06 (t, J = 9.2 Hz, 1H), 2.98 (t, J = 8.8 Hz, 1H) ppm.

Compound **c**: Diethyl malonate (0.5 mL, 3.25 mmol) and piperidine (0.2 mL) were added to 4-(diethyl amino) salicylic aldehyde (500 mg, 2.5 mmol) ethanol (1.7 mL) solution. The reaction mixture was stirred at room temperature for 2 hours. Then the mixture was diluted with water (25 mL) and extracted with ethyl acetate (25 mL × 3). The combined organic phase was dried over Na_2SO_4 and evaporated to dryness. Finally, **c** was purified by flash column chromatography on silica to give yellow solid, yield 95%. ^1H NMR (400 MHz, Chloroform-*d*) δ 8.42 (s, 1H), 7.35 (d, J = 9.0 Hz, 1H), 6.60 (dd, J = 8.9, 2.5

Hz, 1H), 6.46 (d, *J* = 2.4 Hz, 1H), 4.36 (q, *J* = 7.1 Hz, 2H), 3.44 (q, *J* = 7.2 Hz, 4H), 1.38 (t, *J* = 7.1 Hz, 3H), 1.23 (t, *J* = 7.2 Hz, 6H) ppm.

Compound **d**: **c** (464.5 mg, 1.6 mmol) was dissolved in anhydrous EtOH (5 mL). To this stirring solution, hydrazine monohydrate (0.311 mL, 6.4 mmol) was added. The reaction mixture was stirred at room temperature until no further precipitate was observed, at which point it was cooled to 0 °C and stirred for another 15 min. The precipitate was collected *via* vacuum filtration and purified by flash column chromatography on silica to give yellow solid, yield 83%. ^{1}H NMR (400 MHz, Chloroform-*d*) δ 9.73 (s, 1H), 8.67 (s, 1H), 7.43 (d, *J* = 8.9 Hz, 1H), 6.65 (dd, *J* = 9.0, 2.5 Hz, 1H), 6.49 (d, *J* = 2.4 Hz, 1H), 3.45 (q, *J* = 7.1 Hz, 4H), 1.24 (t, *J* = 7.1 Hz, 6H) ppm.

Compound **e**: Tetra (ethylene glycol) (9.78 g, 50.4 mmol) was dissolved in THF (20 mL) followed by adding NaH (1.205 g, 25.2 mmol) at 0 °C. Then 3-bromopropyne (0.885 g, 7.425 mmol) was dissolved in THF (3.75 mL) and added dropwise into the mixture at 0 °C. The reaction mixture was stirred at room temperature for 2 hours, then diluted with water (25 mL). The THF was evaporated in vacuum. The aqueous solution was extracted with dichloromethane (50 mL × 3). The combined organic phase was dried over Na_2SO_4 and evaporated to dryness to give pale yellow oil, yield 88%. ^{1}H NMR (400 MHz, Chloroform-*d*) δ 4.20 (d, J = 2.4 Hz, 2H), 3.74 – 3.64 (m, 15H), 3.60 (dd, *J* = 5.3, 3.5 Hz, 2H), 2.43 (d, *J* = 4.8 Hz, 1H) ppm.

Compound **f**: NaOH (1.0 g, 25 mmol) in water (10 mL) was added into the solution of **e** (1.995 g, 8.6 mmol) and Tosyl chloride (2.767 g, 14.53 mmol) in THF (24 mL) under 0 °C. The reaction mixture was stirred at room temperature for 2.5 hours and then evaporated in vacuum. The crude product was purified by flash column chromatography on silica to give colorless oil, yield 94%. ^{1}H NMR (400 MHz, Chloroform-*d*) δ 7.84 – 7.75 (m, 2H), 7.33 (d, *J* = 8.0 Hz, 2H), 4.19 (d, *J* = 2.4 Hz, 2H), 4.17 – 4.12 (m, 2H), 3.73 – 3.56 (m, 14H), 2.44 (s, 3H), 2.42 (t, *J* = 2.4 Hz, 1H) ppm.

Compound **g**: **f** (0.775 g, 2.0 mmol), imidazole-2-carboxaldehyde (211.5 mg, 2.2 mmol), and K_2CO_3 (414.5 mg, 3.0 mmol) were added into dry DMF (10 mL). The reaction mixture was refluxed for 18 hours under 110 °C and then diluted with water (10 mL). The mixture was extracted with ethyl acetate and the combined organic phase was evaporated in vacuum. The crude product was purified by flash column chromatography on silica to give colorless oil, yield 61%. ^{1}H NMR (500 MHz, Chloroform-*d*) δ 9.76 (s, 1H), 7.32 (s, 1H), 7.22 (s, 1H), 4.56 (t, *J* = 5.0 Hz, 2H), 4.15 (d, *J* = 2.2 Hz, 2H), 3.74 (t, *J* = 5.0 Hz, 2H), 3.69 – 3.61 (m, 4H), 3.61 – 3.56 (m, 4H), 3.54 (s, 4H), 2.40 (t, *J* = 2.5 Hz, 1H) ppm.

Compound **h**: The solution of $CuSO_4{\cdot}5H_2O$ (26 mg, 0.1 mmol) and sodium ascorbate (104 mg, 0.35 mmol) in water (2 mL) was added into the solution of **b** (119.5 mg，0.6 mmol) and **g** (296.5 mg, 0.95 mmol) in water (7 mL) and THF (6 mL). The reaction mixture was stirred at room temperature for 24 hours and then evaporated in vacuum. The crude product was purified by flash column chromatography on silica to give colorless oil, yield 45%. ^{1}H NMR (400 MHz, DMSO-d_6) δ 9.68 (s, 1H), 8.27 (s, 1H), 7.62 (s, 1H), 7.26 (s, 1H), 5.51 (d, *J* = 9.3 Hz, 1H), 5.44 (d, *J* = 6.1 Hz, 1H), 5.32 (d, *J* = 4.9 Hz, 1H), 5.21 (d, *J* = 5.6 Hz, 1H), 4.72 (t, *J* = 5.6 Hz, 1H), 4.58 – 4.43 (m, 4H), 4.17 (s, 6H), 3.93 – 3.60 (m, 18H), 3.27 – 3.20 (m, 2H) ppm.

GluC: **h** (121.5 mg, 0.25 mmol) and **d** (68 mg, 0.25 mmol) were dissolved in methanol (4 mL) followed by adding a drop of acetic acid. The reaction mixture was stirred at room temperature for 6 hours and then evaporated in vacuum. The crude product was purified by flash column chromatography on silica to give yellow solid, yield 91%. ^{1}H NMR (400 MHz, Chloroform-*d*) δ 14.82 (s, 1H), 8.65 (d, *J* = 21.1 Hz, 1H), 8.10 (s, 1H), 7.73 – 7.34 (m, 2H), 7.32 – 7.22 (m, 1H), 7.09 (s, 1H), 6.58 (t, *J* = 10.9 Hz, 1H), 6.45 – 6.29 (m, 1H), 5.72 (s, 1H), 4.50 (s, 4H), 4.14 (d, *J* = 76.6 Hz, 4H), 3.80 (d, *J* = 36.8 Hz, 9H), 3.58 – 3.20 (m, 15H), 1.19 (t, *J* = 6.8 Hz, 6H) ppm.

Cu[GluC]: $Cu(ClO_4)_2{\cdot}6H_2O$ (37 mg，0.1 mmol) and GluC (77 mg，0.1 mmol) were dissolved in methanol (15 mL). The reaction mixture was refluxed for 2 hours under 80 °C

and then evaporated in vacuum to give crimson solid, stoichiometrically. MS (ESI) $C_{35}H_{47}N_8O_{12}Cu^{2+}$ calc. for [M], 834.2604, found 834.2602.

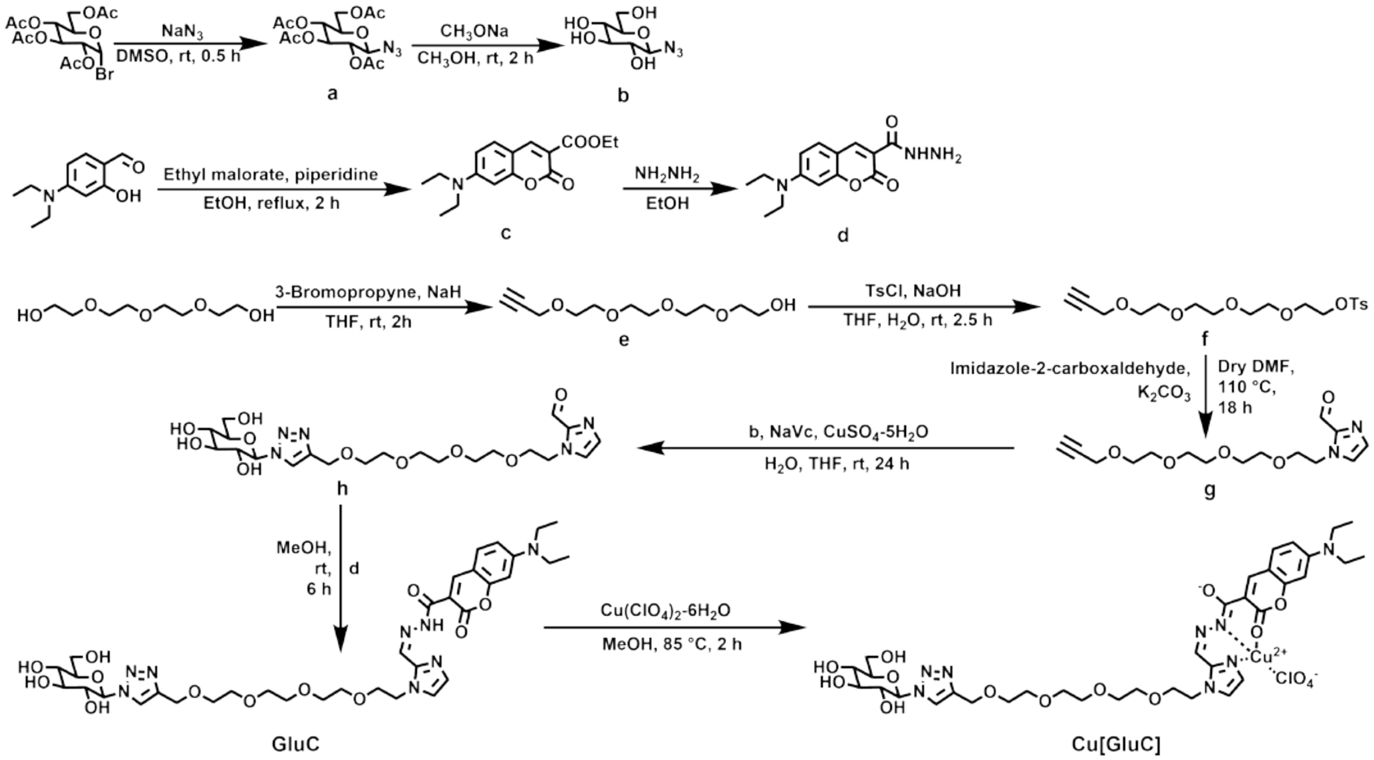

Scheme S1. Synthesis of bioprobe Cu[GluC].

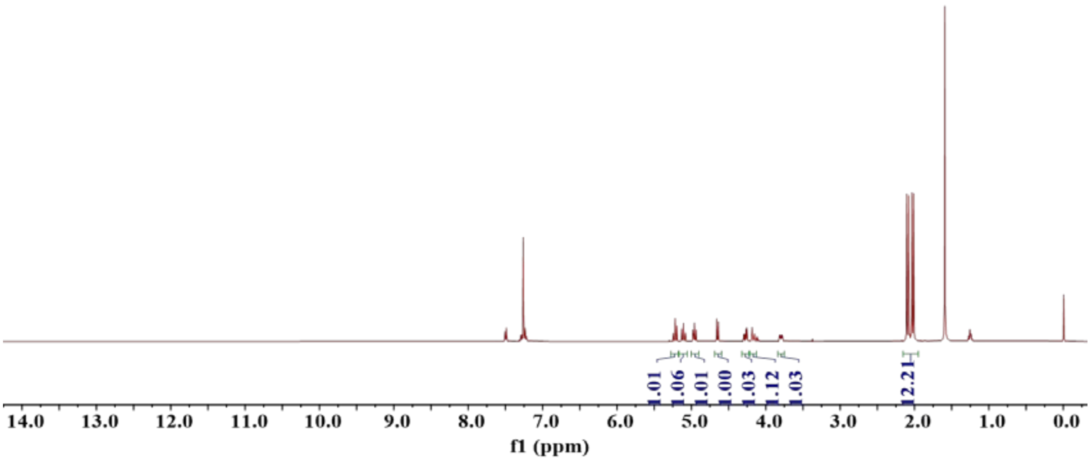

Figure S1. ^{1}H NMR spectrum of compound **a** in Chloroform-*d*.

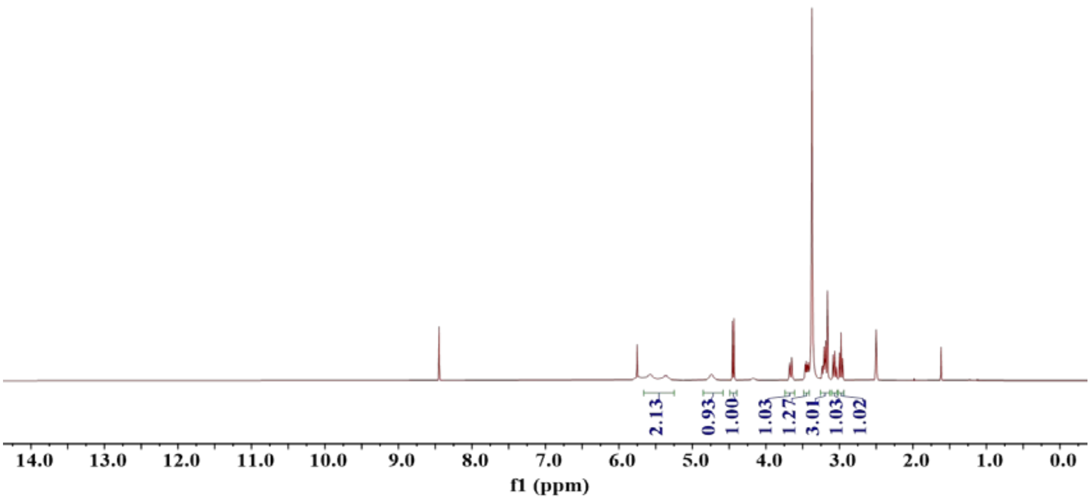

Figure S2. ^{1}H NMR spectrum of compound **b** in DMSO-*d*6.

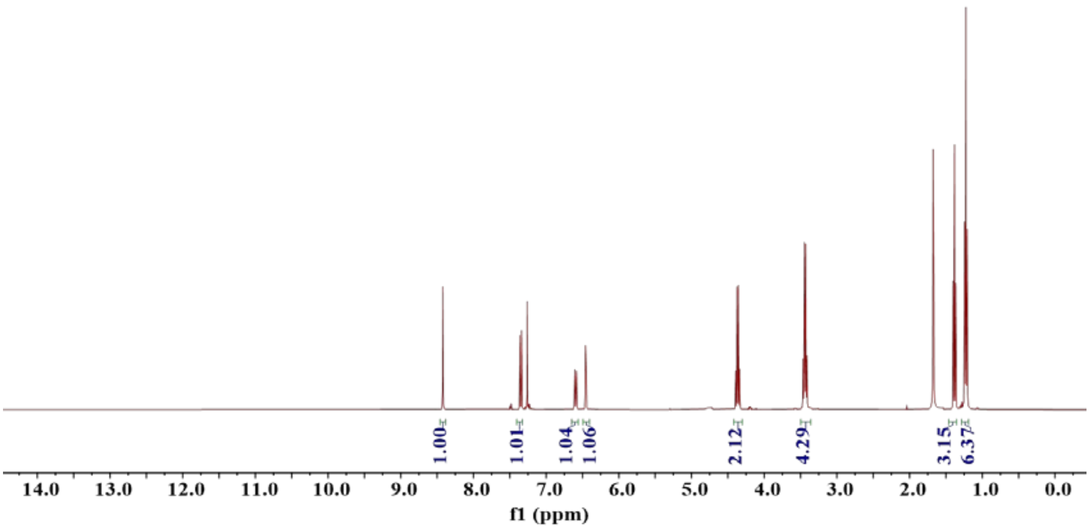

Figure S3. ^{1}H NMR spectrum of compound **c** in Chloroform-*d*.

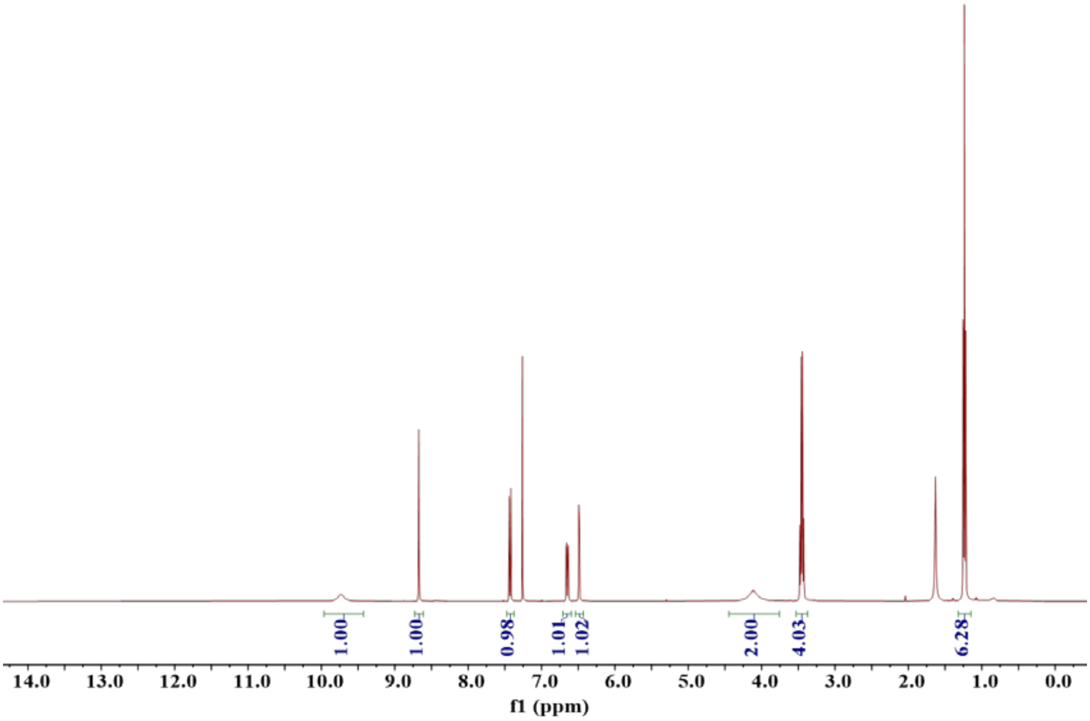

Figure S4. ^{1}H NMR spectrum of compound **d** in Chloroform-*d*.

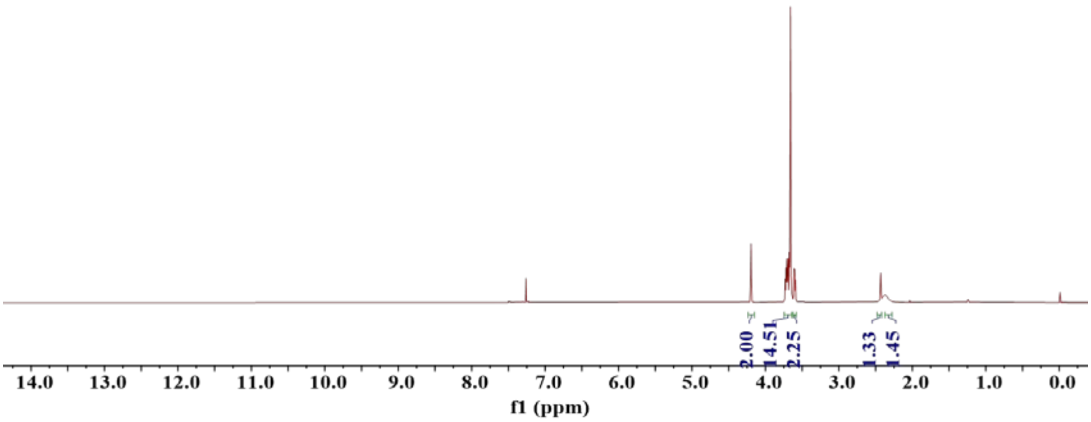

Figure S5. ^{1}H NMR spectrum of compound **e** in Chloroform-*d*.

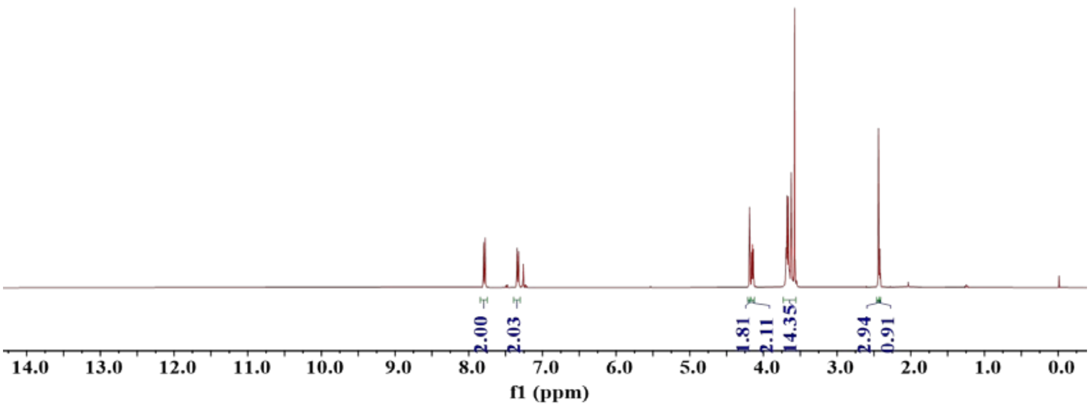

Figure S6. ^{1}H NMR spectrum of compound **f** in Chloroform-*d*.

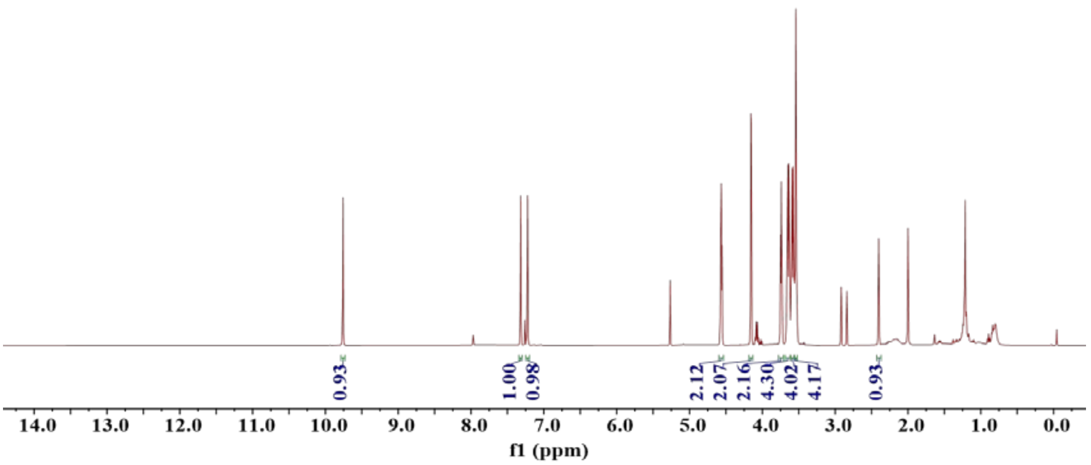

Figure S7. ^{1}H NMR spectrum of compound **g** in Chloroform-*d*.

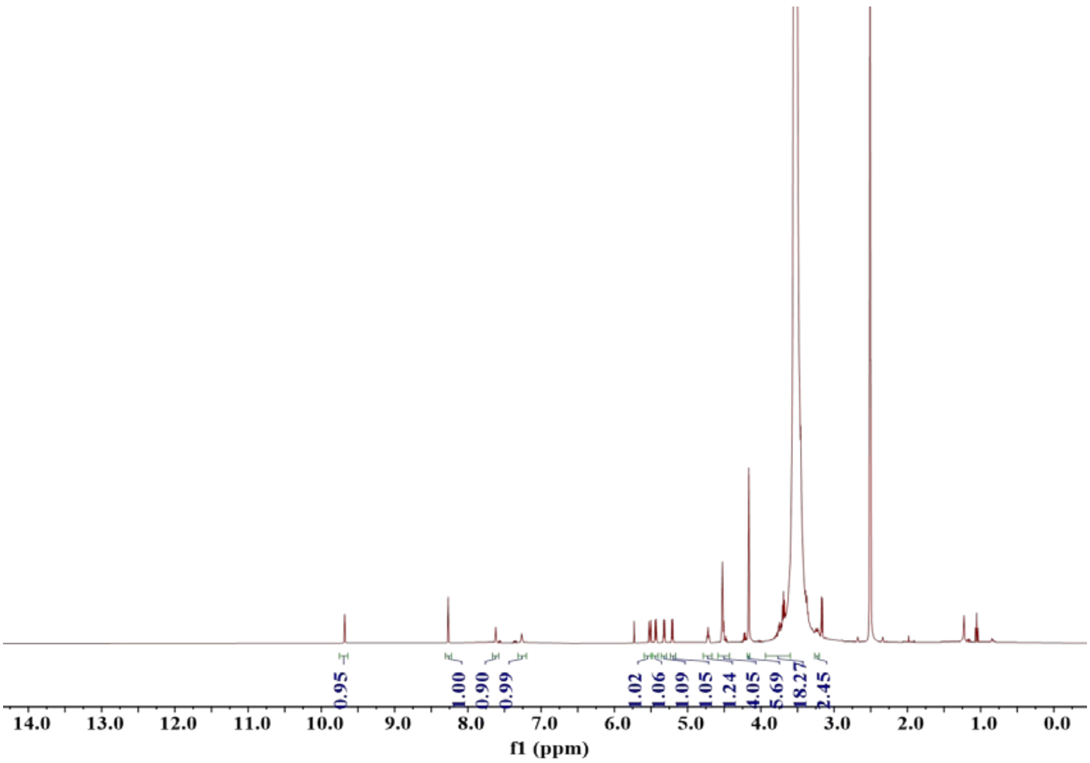

Figure S8. ^{1}H NMR spectrum of compound **h** in DMSO-*d*6.

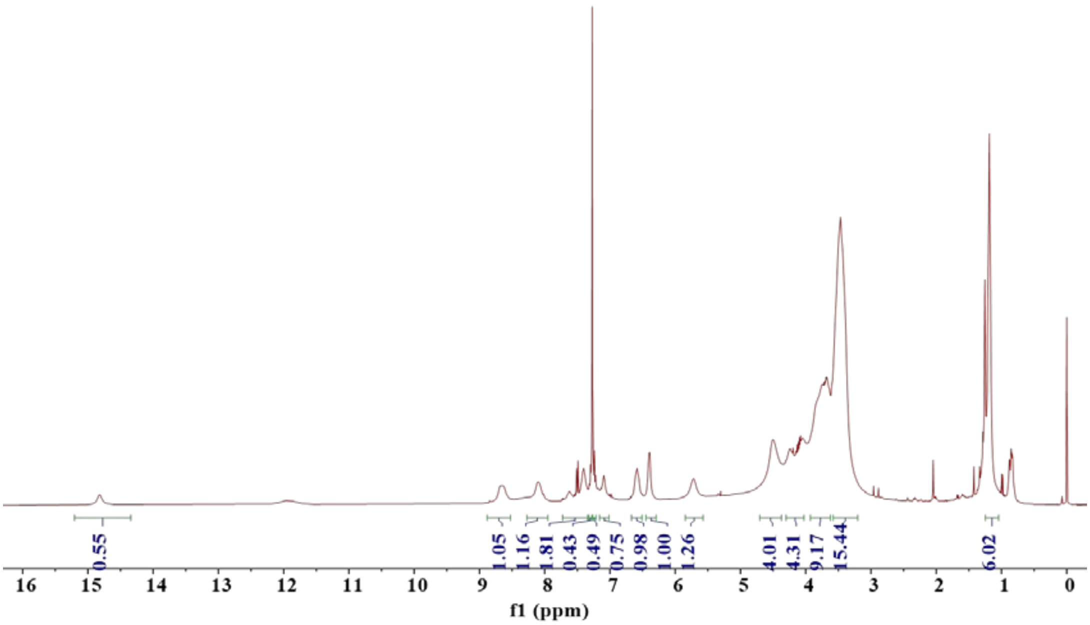

Figure S9. ^{1}H NMR spectrum of GluC in Chloroform-*d*.

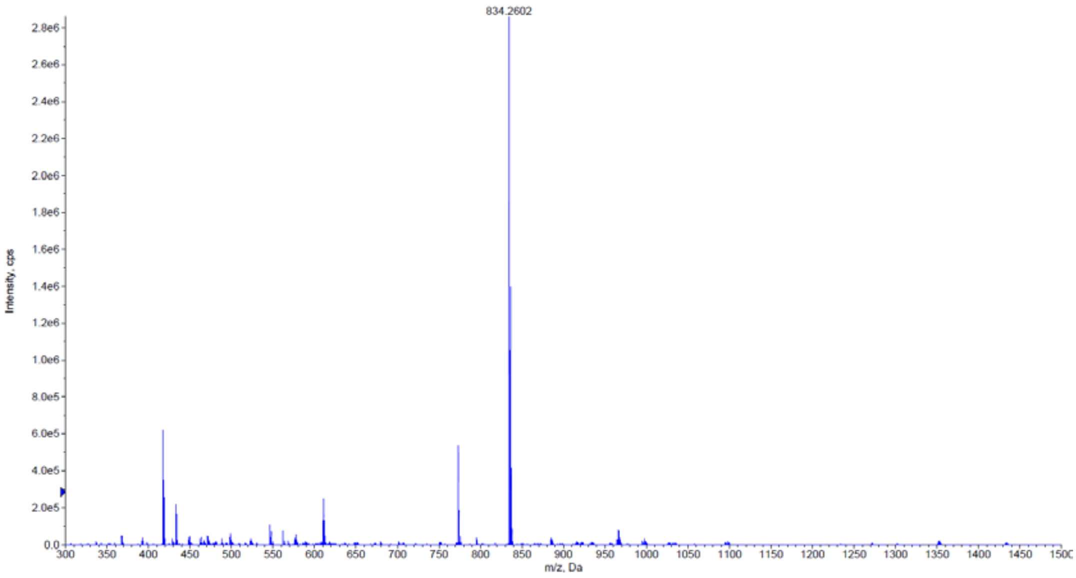

Figure S10. HRMS spectrum of Cu[GluC].

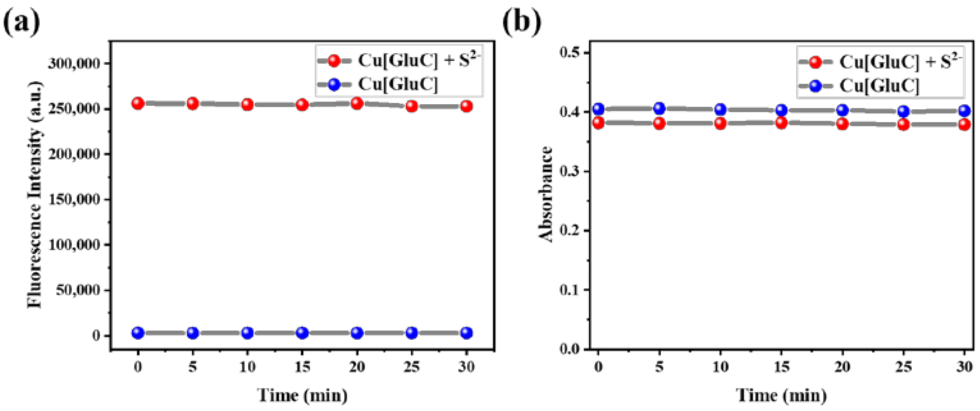

Figure S11. The fluorescence intensity (at 494 nm) (**a**) and absorbance (at 460 nm) (**b**)variation process of Cu[GluC] (10.0 μM) and Cu[GluC] (10.0 μM) + S^{2-} (30.0 μM) in water within 30 min.

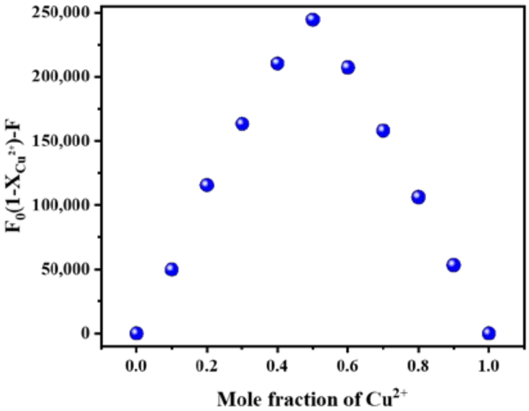

Figure S12. Job's plot for the stoichiometry of GluC and Cu^{2+} ions. (The sum of Cu^{2+} and GluC concentrations in the Job's experiment is 10 μM.)

Table S1. Some reported fluorescent probes for S^{2-} detection in water.

**Structure of probe**	**LOD**	**Solvent**	**pH range**	**Response time**	**Highlights**	**Reference**
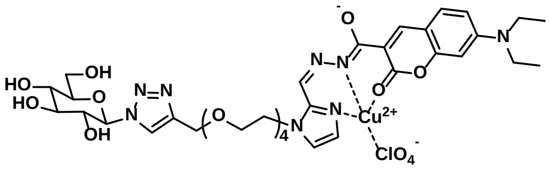	49.6 nM	water	6.0-11.0	20 s	A water-soluble fluorescent bioprobe and its derived fluorescent test strip for selective and sensitive detection of S^{2-} in water	This work
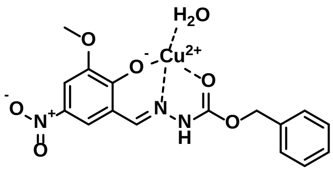	0.6 μM	near-perfect water	6.0-9.0	/	Selective detection of Cu^{2+} and S^{2-} with low detection limits	[1]
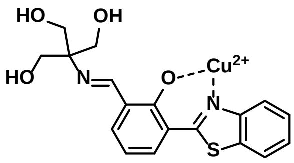	0.3 μM	water	5.8-8.0	2 min	1) Reversible detection of Cu^{2+} and S^{2-} in 100% aqueous solution. 2) Imaging of hydrogen sulfide both in living cells.	[2]
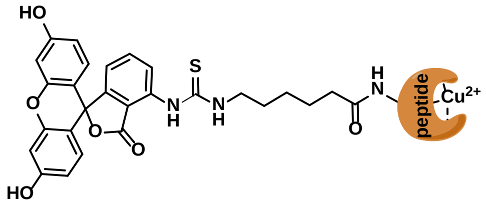	31 nM	water	7.0-10.0	/	Selective imaging of hydrogen sulfide both in living cells and zebrafish	[3]
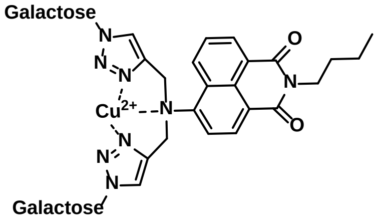	3.2 nM	water	5.0-11.0	/	1) Selective detection of S^{2-} through a displacement protocol in full aqueous solution. 2) Quantification of S^{2-} in real water samples.	[4]

References

1. Park, S.; Choe, D.; Lee, J.J.; Kim, C. A benzyl carbazate-based colorimetric chemosensor for relay detection of Cu^{2+} and S^{2-} in near-perfect aqueous media. *J. Mol. Struct.* **2021**, *1240*, 130576.
2. Wang, H.; Shi, D.-L.; Li, J.; Tang, H.-Y.; Li, J.; Guo, Y. A facile fluorescent probe with a large Stokes shift for sequentially detecting copper and sulfide in 100% aqueous solution and imaging them in living cells. *Sens. Actuators B Chem.* **2018**, *256*, 600–608.
3. Wang, P.; Wu, J.; Di, C.; Zhou, R.; Zhang, H.; Su, P.; Xu, C.; Zhou, P.; Ge, Y.; et al. A novel peptide-based fluorescence chemosensor for selective imaging of hydrogen sulfide both in living cells and zebrafish. *Biosens. Bioelectron.* **2017**, *92*, 602–609.
4. Li, K.-B.; Jia, W.-P.; Han, D.-M.; Liang, D.-X.; He, X.-P.; Chen, G.-R. Fluorogenic bis-triazolyl galactoprobe–metal complex for full-aqueous analysis of sulfide ion. *Sens. Actuators B Chem.* **2017**, *246*, 197–201.
